# Huang Qi Tong Bi Decoction Attenuates Myocardial Ischemia-Reperfusion Injury via HMGB1/TLR/NF-*κ*B Pathway

**DOI:** 10.1155/2019/8387636

**Published:** 2019-03-03

**Authors:** Kun Liu, Manman Li, Xiumei Ren, Qing-sheng You, Fei Wang, Shuo Wang, Chun-Hui Ma, Wei-Nan Li, Qing Ye

**Affiliations:** ^1^Department of Cardiothoracic Surgery, Affiliated Hospital of Nantong University, Nantong, China; ^2^School of Life Science and Technology, China Pharmaceutical University, Nanjing 210009, China; ^3^Department of Traditional Chinese Medicine, Affiliated Hospital of Nantong University, Nantong, China; ^4^Department of Obstetrics and Gynecology, Affiliated Hospital of Nantong University, Nantong, China

## Abstract

The aim of this study was to study the protective effect of Huang Qi Tong Bi Decoction (HQTBT) on the heart of rats. Ischemia-reperfusion injury was established by coronary artery ligation. Proinflammatory cytokines were decreased by XFZY in coronary artery ligated rats. ST segment was also restored with the treatment of HQTBT. Triphenyltetrazole chloride (TTC) staining and pathological analysis showed that HQTBT reduced myocardial injury. Besides, the expressions of HMGB1/TLR/NF-*κ*B pathway in rats were significantly decreased by HQTBT. This study shows that HQTBT inhibited inflammatory reaction on myocardial injury in rats.

## 1. Introduction

Myocardial ischemia-reperfusion injury (I/R) is a common and inevitable problem in the era of coronary intervention. It has a great impact on the quality of life of patients after intervention. Therefore, it is of great significance to the pathogenesis and pathophysiological changes of myocardial ischemia-reperfusion injury. More and more attention has been paid to the follow-up treatment of reconstruction of blood supply for patients in cardiosurgery and cardiology, although there is no mechanism that can perfectly explain the pathogenesis of myocardial ischemia-reperfusion injury [1, 2]. China has the world's second largest population, and its population is aging gradually. Besides tumors, coronary heart disease has become a common disease and cause of death among the elderly, with the incidence increasing year by year. It has become particularly urgent and important to explore the treatment and prevention of myocardial ischemia. In addition to intervention to solve the problem of large blood vessels in the heart, drug therapy has become the main method to treat coronary heart disease because cardiovascular intervention is only an emergency treatment to restore the blood supply to the heart, and the treatment shortage of subsequent ischemia-reperfusion injury is a heart microworld disease that cannot be reached by modern cardiovascular intervention medical conditions, accounting for more than 90% of the heart vascular disease. This 90% heart microworld has become the focus of research in modern cardiology [3, 4].

Inflammation is the key to host defense against invading pathogens. In response to infection, communication of signals leads to recruitment of neutrophils and macrophages, phagocytosis of innate immune cells of infectious organisms, and production of other cytokines and chemical factors leading to lymphocyte activation and adaptive immune response. Inflammatory reactions are also essential for the repair of tissues and wounds. Inflammation can also be caused by I/R, which usually occurs in the absence of microorganisms and is therefore called aseptic inflammation. Similar to the response of invasive pathogens, I/R-induced aseptic inflammation is characterized by neutrophil recruitment and the production of cytokines, chemokines, and other proinflammatory stimuli. Activated neutrophils infiltrate ischemic tissues to produce ROS, release hydrolase, and secrete pore-forming molecules, resulting in extensive damage to parenchymal cells [5–7].

Due to centuries of experience in clinical practice and the fact that compounds derived from traditional Chinese medicines have many targets, instead of using a single compound/single target drug discovery paradigm [8], more and more studies show that traditional Chinese medicine is a potentially powerful new drug. Huang Qi Tong Bi Decoction (HQTBT) is an ancient traditional Chinese medicine formula for treating coronary heart disease in clinic [9]. However, the mechanism of HQTBT on cardioprotection is not well explained. Therefore, the present study evaluated the pharmacological effects of HQTBT on I/R-induced heart injury and investigated its potential mechanism.

## 2. Methods

### 2.1. HQTBT Decoction

Astragalus membranaceus (Fisch.) Bunge, Radix Angelicae Sinensis, Radix Paeoniae Alba, Ligusticum chuanxiong Hort, Rehmannia glutinosa Libosch, and Glycyrrhiza uralensis Fisch were prepared for crude plant medicines from Nantong Tong Ren Tang pharmacy. Each herb was authenticated by the herbal medicinal botanist, Professor Wei Zhang, at the Department of Pharmacy of Nantong University. According to the standard process, HQTBT dried powder was produced using 11 HQTBT herbs of the above ratio. According to the standard of 1 g/ml (w/v), distilled water is stored at 4°C before being dissolved for use.

### 2.2. HPLC Analysis of HQTBT Decoction

HPLC was performed using Agilent 1200 HPLC with G1321A FD and Eclipse AAA column (4.6 × 150 mm, 5 *μ*m) and a column temperature of 40°C: mobile phase A (formic acid : water = 1 : 1000) and mobile phase B (acetonitrile). The gradient elution procedure was 0–13 minutes, B (0%–63%), 0–8 minutes, B (63%), 9-10 minutes, B (100%), 11 minutes, B (100%–0%), and 12-13 minutes, B (0%). The flow rate was 0.4 ml min^−1^.

### 2.3. Reagents

The kits of MDA and SOD were purchased from Jiancheng Bioengineering Institute (Nanjing, China). IL-6, IL-1*β*, and TNF-*α* ELISA kits were produced by Nanjing KeyGen Biotech. Co. Ltd. (Nanjing, China). All the antibodies were provided by Cell Signaling Technology (Danvers, USA).

### 2.4. Animals

Healthy male SD rats, weighing 250-300 g, were purchased from Nantong University. The rats were kept in polypropylene cages at a temperature of 25 ± 0.5°C and a relative humidity of 60 ± 5 <UNK> and 12 hours/12 small time/dark cycle. All animals follow an internationally accepted code of ethics of Nantong University.

### 2.5. Experimental Protocol of the Left Coronary Artery Ligation

The procedures for the left coronary artery ligation are as follows: first, a rat muscle injection of 10 mg/kg serazine hydrochloride and 100 mg/kg ketamine anesthesia, limb II guide electrocardiogram. The body temperature is maintained at 37.0 ± 0.5°C. Next is tracheotomy intubation, connecting small animal ventilators (frequency 60 times per minute, pour/aspiration = 1 : 1.5, tidal volume 3 ml/100 g body weight, 60 inches oxygen). The ventilation was adjusted by repeated arterial blood gas analysis throughout the experiment. Use a polyethylene tube to intubate the right common carotid artery to take blood samples and measure arterial blood pressure and heart rate. Artery intubation is connected to pressure sensors. Continuous monitoring of average blood pressure (BP) and heart rate (HR) is done. Cut the skin and subcutaneous tissue 0.5 cm along the left side of the sternum, separating the chest muscles and fascia, about 3 to 4 cm long, and blunt separation of intercostal muscles about 3 cm long between the fourth left ribs, cutting the fourth. Open the chest, find the large heart vein associated with the left coronary artery between the left heart ear and the pulmonary artery cone, 4-0 surgical wire noninvasive round needle injection needle at 2 mm below the base of the left heart ear, pulmonary artery Panicle needle, injection depth 1-1.5 mm, 2 to 3 mm wide, combined with concentric large veins, ligation of the left coronary artery anterior descending branch (false surgical group only thread not ligation). The reversible occlusion of the left aorta descending coronary artery after pericardiotomy was prepared by ligation of the ends with a vinyl tube. After 30 minutes of local ischemia, the trap is set loose and the myocardium underwent reperfusion for 120 minutes. After ligation of the left anterior descending branch, the left ventricular anterior wall myocardium became white, the local contraction movement was limited, the left heart ear was full, and the electrocardiogram shows that the ST segment continued to raise the bow back, indicating that the myocardial ischemia was successful. Untie the ligation line, see the heart surface from pale to red, electrocardiogram ST segment raised part fell, and judge the success of reperfusion [10]. The rats were divided into 4 groups as follows: sham group, model group (I/R), I/R+HQTBT (6 g/kg), and I/R+HQTBT (12 g/kg). HQTBT was given for 7 days after surgery. 24 h after the operation, the blood samples were collected and centrifuged at 3500 g for 15 min, and the supernatant was stored at −80°C for further analysis. Then the hearts of the rats were collected for other analysis.

### 2.6. Electrocardiogram

The ECG of I/R rats was obtained in standard lead II using a computerized power laboratory system, and the ST segment elevation was measured at 5 min before ligation, 30 min after ligation, and 120 min after reperfusion of the left anterior descending branch of ischemia/reperfusion rats.

### 2.7. Determination of Myocardial Infarct Size

Quickly take out the rat heart, wash away blood stains in saline water placed on ice, remove irrelevant tissues above the ligature with a blade, then freeze the heart in a -20°C refrigerator for 30 min, place it in a 1% TTC solution, and stand in 37°C water for 15 min in the dark.

### 2.8. Determination of Inflammatory Cytokines in the Serum and Heart

The rats were fixed on the rat table, cut through with scissors along the midline of the abdomen, slowly separated the peritoneum to both sides with cotton balls, opened the viscera to expose the abdominal aorta, collected 5 ml of blood with a 10 ml syringe, coagulated for 20 minutes under natural conditions, centrifuged the blood at 4°C (3000 r/min), and put the supernatant into EP tubes after 15 min. If it could not be detected in time, it was stored in -80°C refrigerator. Detect the levels of IL-6, IL-1*β*, and TNF-*α* in serum with ELISA kit according to instructions.

### 2.9. Determination of Cardiac Marker Enzymes in the Serum and Heart

The rats were fixed on the rat table, cut through with scissors along the midline of the abdomen, slowly separated the peritoneum to both sides with cotton balls, opened the viscera to expose the abdominal aorta, collected 5 ml of blood with a 10 ml syringe, coagulated for 20 minutes under natural conditions, centrifuged the blood at 4°C (3000 r/min), and put the supernatant into EP tubes after 15 min. If it could not be detected in time, it was stored in -80°C refrigerator. Detect the levels of CK and LDH in the serum and heart with commercial kits according to instructions.

### 2.10. Histological Examination of the Myocardium

The heart tissue was carefully cut into slices of about 4 mm using a cryoslicer. Put the cut tissue in the fixing solution for about 10-30 s, and wash the L (2s) in water after fixing. After that, it was dyed with hematoxylin for 0.5 min and washed with running water. Then, 1% hydrochloric acid alcohol was used to differentiate 1-3 s, weak ammonia water was used to soak for 5-10 s, 0.5% eosin solution was used to dye for 30-60 s, and ethanol with different concentrations was used to wash for 2 s in turn. The final gum (formulated as neutral) seal was observed under an optical microscope.

### 2.11. Western Blot

The total protein of the heart tissue was extracted with RIPA lysate, centrifuged at 12,000 rmin^−1^ for 15 min, and the supernatant was collected. The BCA kit was used for protein quantification. The protein sample was placed in SDS-polyacrylamide gel electrophoresis until the sample reached the bottom of the separation gel and transferred to PVDF membrane. After the film transfer was completed, the strips were immersed in 5% skimmed milk and sealed for 2 h. After closing, the PVDF membranes were immersed in the primary antibody and incubated overnight at 4°C. On the second day, the first antibodies were discarded and the second antibody was added to incubate at room temperature for 2 h. After the second antibody was incubated, the antibody was discarded and the PVDF membrane was removed and washed three times with TBST. The gel imaging system is used for exposure and the gel scanning imaging system is used for scanning the bands to analyze the gray value of each band.

### 2.12. Immunohistochemical Analysis

Immunohistochemical analysis was done as follows:

1. 4% paraformaldehyde was fixed by conventional perfusion, and 20% sucrose solution (4°C) was taken and placed overnight. Wax blocks were made, sliced, and pasted. 0.01 M PBS was used to wash for 5 min × 3
2. Dewaxing and hydration: use xylene twice for 10 min and use gradient ethanol to hydrate from low concentration to high concentration
3. Add 0.3% hydrogen peroxide methanol solution (methanol 80 ml + 0.01 M PBS 100 ml + 30% hydrogen peroxide) for 30 min to eliminate the influence of endogenous peroxidase, and wash with 0.01 M PBS 5 min × 3
4. Add 0.3% Triton X-100 (0.3 ml Triton X-100 + 0.01 M PBS 100 ml) for 30 min to increase cell permeability, and wash with 0.01 M PBS 5 min × 3
5. Add the first antibody diluted with serum diluent (1.00 g of bovine serum albumin + 0.01 M PBS 100 ml + 0.08 g of azide) and store it at 4°C for 24-48 hours. The antibody was aspirated and washed with 0.01 M PBS for 5 min × 3
6. Add 0.01 M PBS diluted secondary antibody and incubate at room temperature for 2 h. 0.01 M PBS was used to wash for 5 min × 3
7. Add antibodies such as ABC complex, incubate at room temperature for 2 h, and wash with 0.01 M PBS for 5 min × 3. The distilled water was quickly used for washing three times
8. Add the color developing solution and carry out immunohistochemical color development for 3-10 min. Observe it under a microscope from time to time. Suck the color developing solution when the cells are colored and the color of the back bottom is light. Rinse it three times with distilled water and then add 0.01 M PBS to stop the reaction
9. Hematoxylin recolor
10. After gradient alcohol dehydration, transparent, sealed and photographed

### 2.13. Statistical Analysis

Data are expressed as standard deviation of average soil, and the normality and homogeneity of variance are tested. The parameters between groups were analyzed by one-way ANOVA and *t*-test. If *p* < 0.05, the difference was statistically significant.

## 3. Results

### 3.1. HPLC Analysis of HQTBT

**Figure 1:**
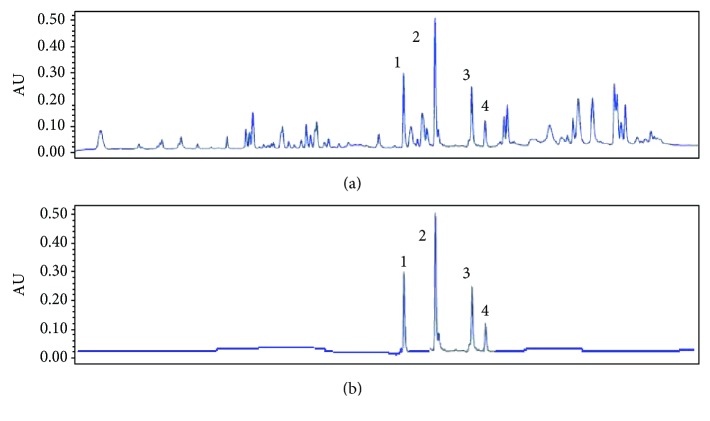
HPLC analysis of HQTBT. (a) HPLC of HQTBT sample. (b) HPLC of standards. 1: astragaloside IV; 2: ferulic acid; 3: ligustrazine; 4: paeoniflorin.

As shown in Figure 1, the contents of amygdalin and hydroxysafflor yellow A have been identified as 0.117 microgram/mg and 0.948 microgram/mg, respectively.

### 3.2. Effect of HQTBT on Myocardial Infarct Size

**Figure 2:**
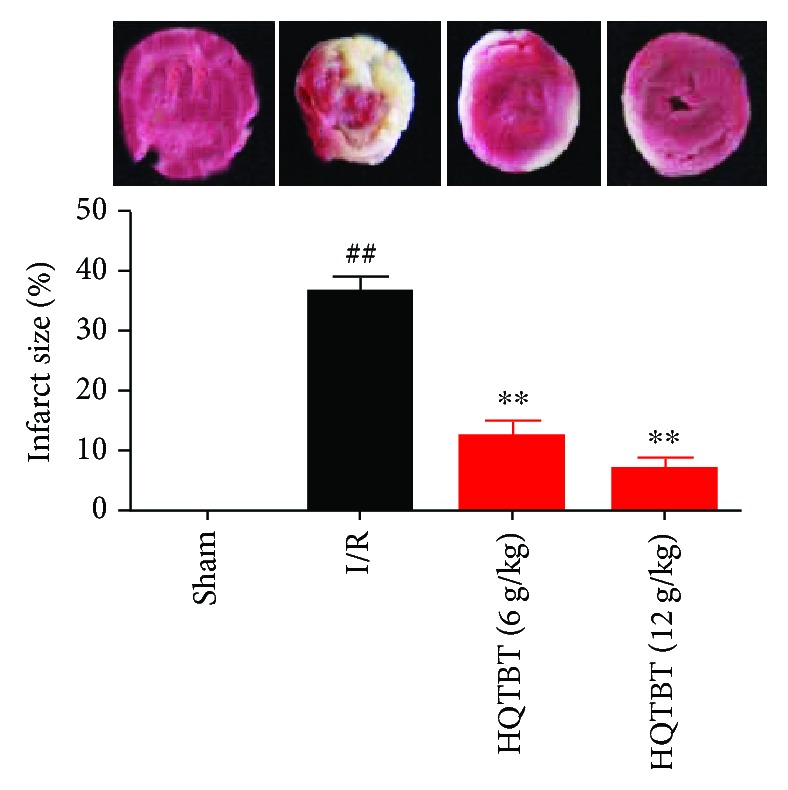
Effect of HQTBT on myocardial infarct size. The data are expressed as mean values ± SDs. ^#^*p* < 0.05, ^##^*p* < 0.01 compared with the control group. ^∗^*p* < 0.05, ^∗∗^*p* < 0.01 compared with the I/R group.

TTC staining is an important indicator of the development of ischemic injury. As shown in Figure 2, the percentage of infarction in the sham operation group is very small, while the area of infarction in the I/R group is larger than that in the sham group. On the contrary, HQTBT treatment significantly reduced the infarct size.

### 3.3. Effect of HQTBT on Myocardial Histology

**Figure 3:**
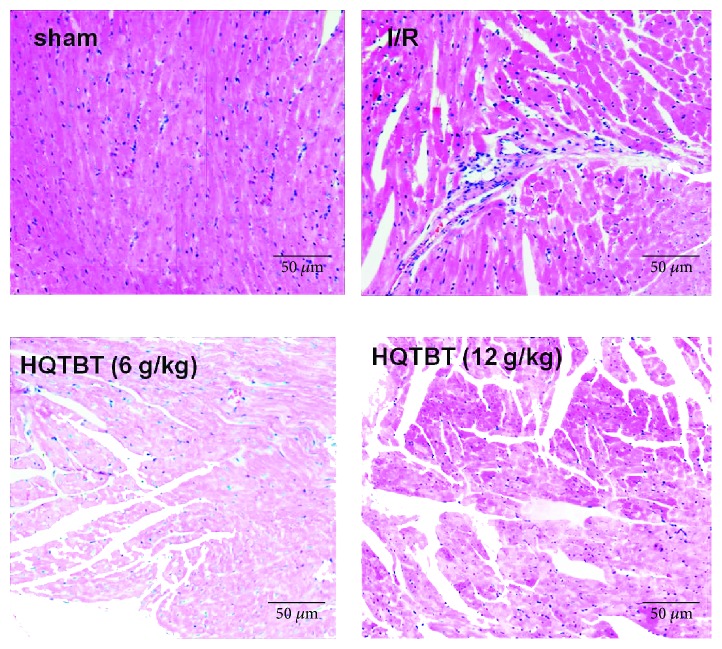
Effect of HQTBT on myocardial histology. Original magnification (×200).

As shown in Figure 3, the tissue of the sham operation group showed a complete myocardial membrane, a well-balanced myofibril structure, and continuous adjacent myofibrils. However, the ligated heart showed a large number of inflammatory cells, swelling or degeneration of myocardial cells, necrosis of the heart, and loss of striations. HQTBT can significantly improve the above pathological changes.

### 3.4. Effects of HQTBT on the ST Segment

**Figure 4:**
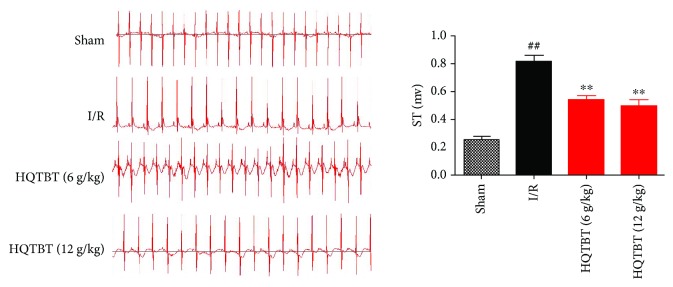
Effects of HQTBT on the ST segment. The data are expressed as mean values ± SDs. ^#^*p* < 0.05, ^##^*p* < 0.01 compared with the control group. ^∗^*p* < 0.05, ^∗∗^*p* < 0.01 compared with the I/R group.

The ECG results are shown in Figure 4. Compared with the control group, the ST segment of the I/R group increased. HQTBT has obviously improved the above phenomenon and partially proved its protective effect.

### 3.5. Effects of HQTBT on Inflammatory Cytokines in the Serum and Heart

**Figure 5:**
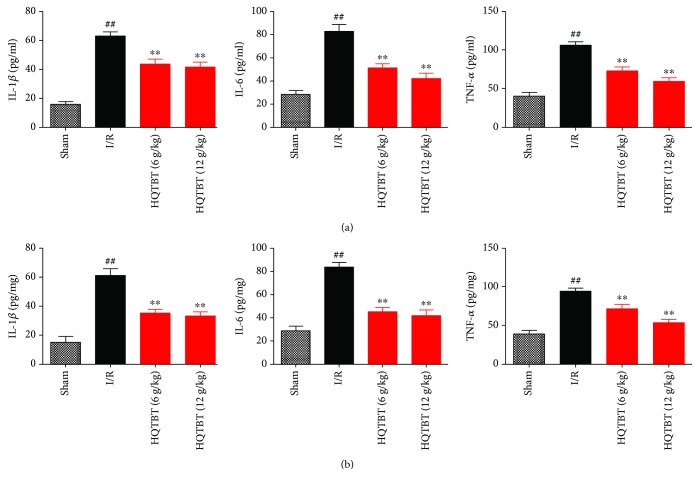
Effects of HQTBT on inflammatory cytokines in the serum and heart. The data are expressed as mean values ± SDs. ^#^*p* < 0.05, ^##^*p* < 0.01 compared with the control group. ^∗^*p* < 0.05, ^∗∗^*p* < 0.01 compared with the I/R group.

Inflammatory cytokines are one of the main indicators of myocardial animal models. The levels of TNF-*α*, IL-6, and IL-1*β* in the serum and heart were increased in I/R rats. Compared with rats in the I/R group, the levels of TNF-*α*, IL-6, and IL-1*β* in the HQTBT treatment group were significantly lower (Figure 5).

### 3.6. Effects of HQTBT on Cardiac Marker Enzymes in the Serum and Heart

**Figure 6:**
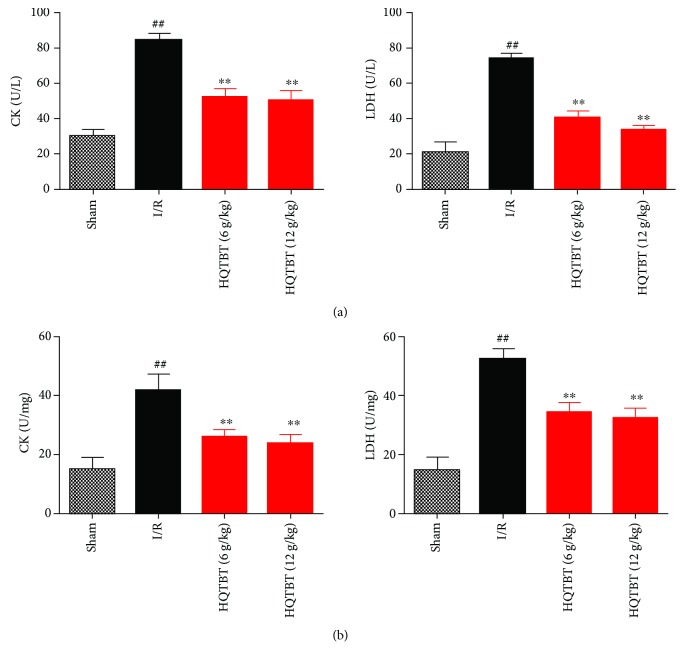
Effects of HQTBT on cardiac marker enzymes in the serum (a) and heart (b). The data are expressed as mean values ± SDs. ^#^*p* < 0.05, ^##^*p* < 0.01 compared with the control group. ^∗^*p* < 0.05, ^∗∗^*p* < 0.01 compared with the I/R group.

The levels of CK and LDH in the serum and heart were measured to detect myocardial injury. As shown in Figure 6, CK and LDH activities in the serum and heart in the I/R group increased significantly compared with those in the sham operation group. As expected, HQTBT significantly reduced CK and LDH levels in the serum and heart compared to the I/R group.

### 3.7. Effects of HQTBT on HMGB1/TLR/NF-*κ*B Pathway in I/R Rats

**Figure 7:**
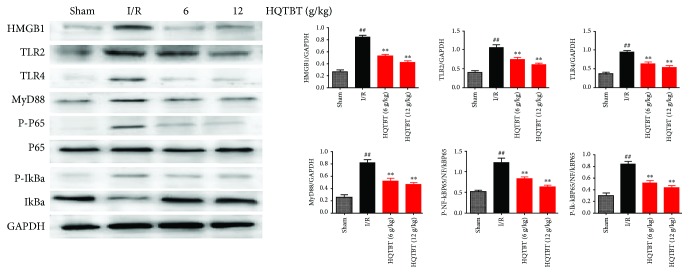
Effects of HQTBT on HMGB1/TLR/NF-*κ*B pathway in I/R rats. The data are expressed as mean values ± SDs. ^#^*p* < 0.05, ^##^*p* < 0.01 compared with the control group. ^∗^*p* < 0.05, ^∗∗^*p* < 0.01 compared with the I/R group.

As shown in Figure 7, HMGB1/TLR/NF-*κ*B pathway was detected in order to clarify the downstream mechanism of HQTBT on I/R-induced rat heart injury. Compared with the sham group, the protein levels of HMGB1, TLR2, TLR4, MyD88, p-IkBa, and p-NF-*κ*Bp65 in the I/R group increased. On the other hand, HQTBT has obvious inhibitory effects on HMGB1, TLR2, TLR4, MyD88, p-IkBa, and p-NF-*κ*Bp65.

### 3.8. Effects of HQTBT on HMGB1 and p-NF-*κ*Bp65 Pathway in I/R Rats

**Figure 8:**
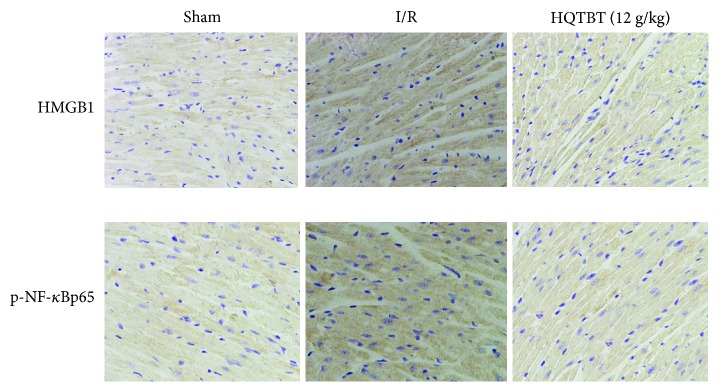
Effects of HQTBT on HMGB1and p-NF-*κ*Bp65 pathway in I/R rats (×200).

As shown in Figure 8, compared with the sham group, the protein levels of HMGB1 and p-NF-*κ*Bp65 in the I/R group increased. On the other hand, HQTBT reduced the levels of HMGB1 and p-NF-*κ*Bp65.

## 4. Discussion

At present, there are more and more studies on ischemia-reperfusion especially on myocardial ischemia-reperfusion injury, which is the main cause of cardiovascular diseases. Cardiovascular diseases have become the leading cause of death worldwide, accounting for 30% of the total number of deaths worldwide. However, myocardial ischemia-reperfusion injury is when the myocardium recovers the blood supply after ischemia, and the myocardium fails to return to its normal function and structure. On the contrary, myocardial cells have been severely damaged. The main clinical symptoms are arrhythmia, myocardial stunning, cardiac dysfunction, myocardial cell injury, and endothelial and microvascular dysfunction. Many medical workers have taken the study of myocardial ischemia-reperfusion injury and its mechanism as their subject. In this study, we observed that HQTBT played a role in myocardial ischemia/reperfusion. In the current research, HQTBT therapy has improved ST, reduced myocardial infarction area, reduced CK-MB and LDH, improved myocardial pathological changes, and restored inflammation-related pathways in myocardial ischemia/reperfusion environment.

The activation of the innate immune system after aseptic injury mainly occurs when the “danger signal” released by damaged cells interacts with the pattern recognition receptor [11]. High-mobility group protein 1 (HMGB1), a highly conserved nuclear protein, affects many biological processes including inflammatory diseases. In addition to nuclear action, it is also released into extracellular fluid as a damage-related molecular model molecule. Extracellular HMGB1 plays a role as a cytokine in inflammation and cell migration, while plasma HMGB1 level is an indicator of disease severity, such as brain, cardiovascular, and renal diseases. Previous studies have shown that extracellular HMGB1 interacts with a variety of cell surface receptors, including toll-like receptor (TLR) 2, TLR 4, or TLR 9, and advanced glycation end product receptor (RAGE), to induce the production of proinflammatory cytokines. In myocardial I/R environment, dangerous signals including heat shock protein (HSPs) are released from the necrotic myocardium and bind to their respective receptors such as TLR4, thus activating the downstream MyD88-NF-*κ*B signaling cascade [12, 13]. The activation of NF-*κ*B is triggered by the phosphorylation and degradation of the I*κ*B*α* [14, 15]. Our results confirmed that HQTBT successfully blocked the expression of HMGB1/TLR/NF-*κ*B pathway.

Myocardial ischemia/reperfusion can cause an increase in the levels of cytokines such as TNF-*α*, IL-6, and IL-1*β*. Infarction increases cell permeability, enhances caspase cascade reaction, and leads to a large number of myocardial cell apoptosis [14]. TNF-*α* is mainly secreted by macrophages, which may promote inflammatory cascade reaction by increasing the release of other proinflammatory cytokines and influencing neutrophil recruitment [15]. IL-6, as an important cytokine in inflammation, plays an important role in inflammation induced by I/R [16]. IL-1*β* can induce the release of other inflammatory mediators, increase the expression of cell adhesion factors, and promote the adhesion of neutrophils to endothelial cells. The experimental results showed that the HQTBT successfully decreased TNF-*α*, IL-6, and IL-1*β* in I/R rats.

In conclusion, in the rat model, HQTBT preconditioning protects the heart from I/R-induced injury. In terms of mechanism, HQTBT reduces the levels of circulating proinflammatory cytokines at least in part by the regulation of HMGB1/TLR/NF-*κ*B signaling pathway. Further research are warranted for more details in the future.

## Data Availability

The data used to support the findings of this study are available from the corresponding author upon request.
